# Supplementary material for: Doping-dependent superconducting physical quantities of K-doped BaFe2As2 obtained through infrared spectroscopy
Source: Sci Rep. 2022 Nov 19;12:19950. doi: 10.1038/s41598-022-24520-y (PMC9675795; doi:10.1038/s41598-022-24520-y)
Supplement: Supplementary file 1 — Supplementary Information. [file 41598_2022_24520_MOESM1_ESM.pdf]

Supplementary Material

Doping-dependent superconducting physical quantities of K-doped $BaFe_2As_2$ obtained through infrared spectroscopy

Seokbae Lee[1], Yu-Seong Seo[1], Seulki Roh[1], Dongjoon Song[2,3], Hiroshi Eisaki[2], and Jungseek Hwang[1,*]

[1]*Department of Physics, Sungkyunkwan University, Suwon, Gyeonggi-do 16419, Republic of Korea*

[2]*National Institute of Advanced Industrial Science and Technology, Tsukuba 305-8568, Japan*

[3]*Department of Physics and Astronomy, Seoul National University, Seoul 08826, Republic of Korea*

DC resistivity data of K-doped Ba-122

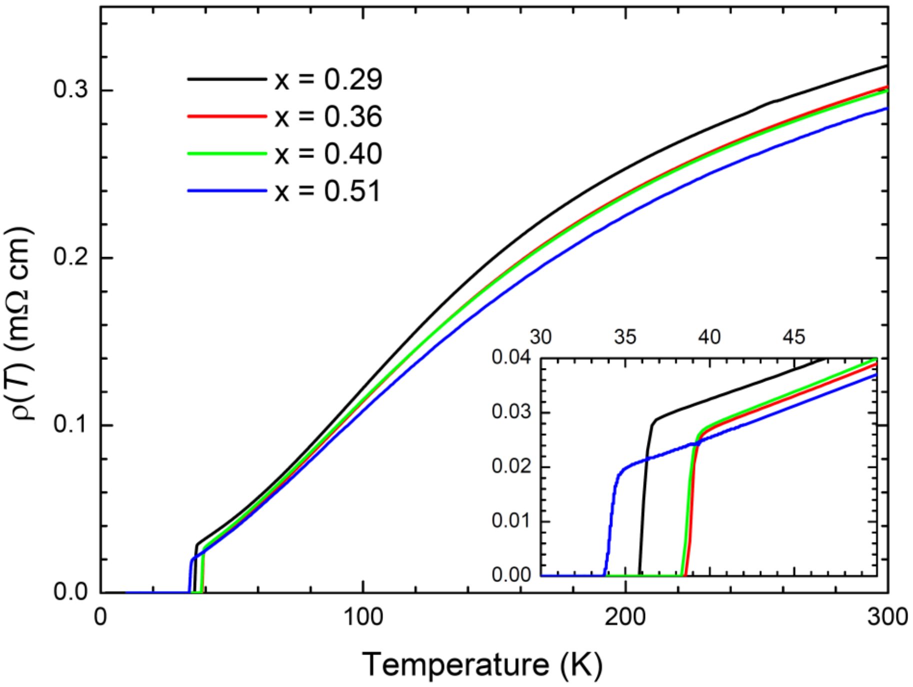

Figure S1. Measured DC resistivity data of K-doped Ba-122 samples are shown. In the inset, a magnified view at low temperatures near the superconducting transition temperatures is shown.

Disorder-dependent superfluid plasma frequency

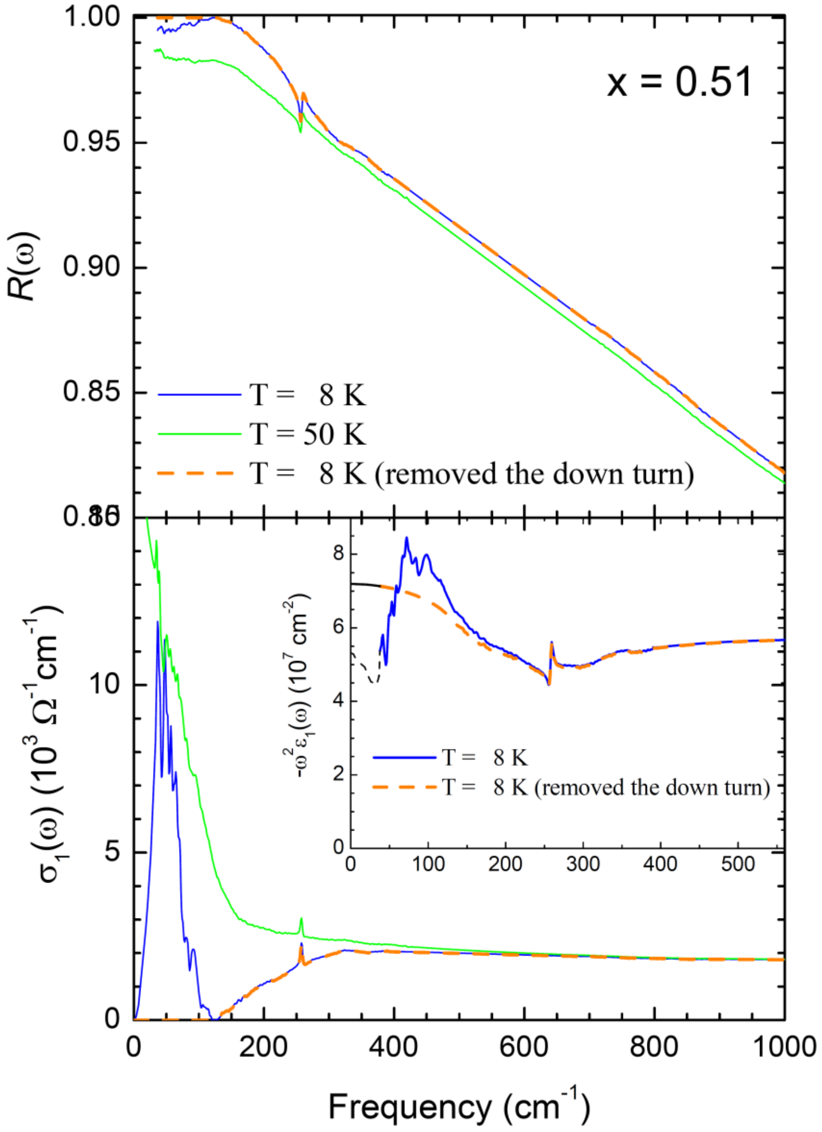

Figure S2. (Upper frame) Measured reflectance spectra of the x = 0.51 sample at 8 K (SC) and 50 K (N) are shown along with a reflectance spectrum at 8 K for the case of removing the down-turn feature below ~120 cm^{-1}, which is caused by the disorder. (Lower frame) Corresponding optical conductivities obtained using the Kramers-Kronig analysis are shown. In the inset, $-\omega^2\varepsilon_1(\omega)$ as functions of w at 8 K for the cases of the original and removing the down-turn feature are shown.

Simulation of the optical conductivity of x = 0.29 sample at 8 K with one channel

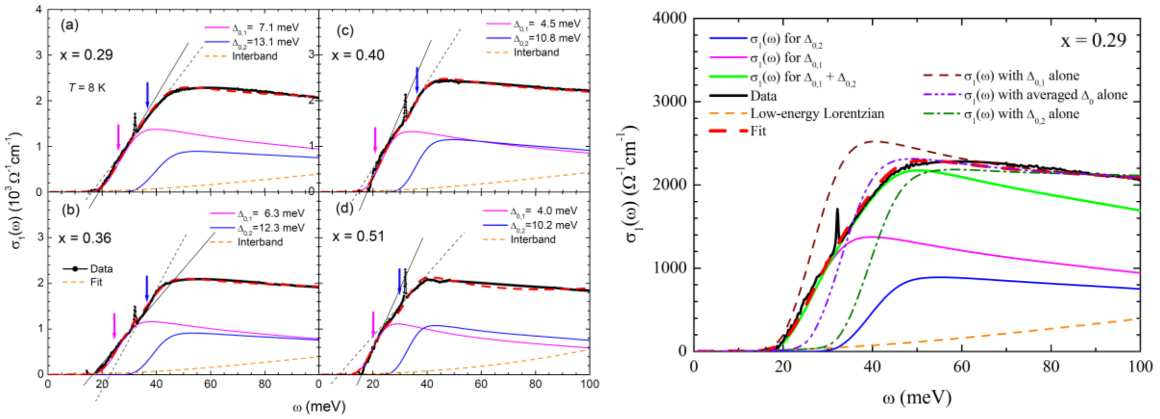

Figure S3. (Left) Change in slope (or kink) in the sharp increase in the optical conductivity. The solid and dashed straight lines are guides to the eyes. (Right) Optical conductivity of the *x* = 0.29 sample at 8 K and fits for a two-parallel-transport-channel and a single-channel approaches. The data cannot be properly simulated with one channel alone in the spectral region below ~50 meV (see Fig. 4a in the main text).
